# Targeting temporal parietal junction for assessing and treating disembodiment phenomena: a systematic review of TMS effect on depersonalization and derealization disorders (DPD) and body illusions

**DOI:** 10.3934/Neuroscience.2021009

**Published:** 2021-01-12

**Authors:** Graziella Orrù, Davide Bertelloni, Valentina Cesari, Ciro Conversano, Angelo Gemignani

**Affiliations:** Department of Surgical, Medical and Molecular Pathology and Critical Care Medicine University of Pisa, via Savi, 10, 56126, Pisa, Italy

**Keywords:** temporal parietal junction, transcranial magnetic stimulation, disembodiment phenomena, depersonalization and derealization disorders, body illusions

## Abstract

The temporal-parietal junction (TPJ) is a key structure for the embodiment, term referred to as the sense of being localized within one's physical body and is a fundamental aspect of the self. On the contrary, the sense of disembodiment, an alteration of one's sense of self or the sense of being localized out of one's physical body, is a prominent feature in specific dissociative disorders, namely depersonalization/derealization disorders (DPD). The aims of the study were to provide: 1) a qualitative synthesis of the effect of Transcranial Magnetic Stimulation (TMS), taking into account its use for therapeutic and experimental purposes; 2) a better understanding on whether the use of TMS could support the treatment of DPD and other clinical conditions in which depersonalization and derealization are displayed. To identify suitable publications, an online search of the PubMed, Cochrane Library, Web of science and Scopus databases was performed using relevant search terms. In addition, an in-depth search was performed by screening review articles and the references section of each included articles. Our search yielded a total of 108 records through multiple databases searching and one additional record was identified through other sources. After duplicates removal, title and abstract reading, we retained 16 records for the assessment of eligibility. According to our inclusion criteria, we retained 8 studies. The selected studies showed that TMS targeting the TPJ is a promising technique for treating disembodiment phenomena DPD and for inducing reversible disembodiment states in healthy subjects. These data represent the first step towards a greater understanding of possible treatments to be used in disembodiment disorders. The use of TMS over the TPJ appears to be promising for treating disembodiment phenomena.

## 1. Introduction

The temporal-parietal junction (TPJ) is a region of the cerebral cortex placed on the border between the temporal and parietal lobes [1]. TPJ is a key hub for a wide range of processes and functions, including: 1) the integration of multisensory-related information [2] and visuospatial perspective taking [3]; 2) self-processing, such as sense of agency [4], self-other distinction and mental own-body imagery [2]; 3) embodiment (the sense of being localized within one's physical body) which is a fundamental aspect of the self [5]. On the contrary, the feeling of disembodiment, as well as the lack of sense of agency or body perceptual distortions/illusions can be experienced in particular states or conditions. The disembodiment is a prominent feature in specific dissociative disorders, namely depersonalization/derealization disorders (DPD). DPD has been estimated to be present in 1–2% of the general population, however empirical evidence suggests that it is severely underdiagnosed [6]. According to DSM-5 [7], DPD is characterized by perceptual alteration in experience toward the integrity of self (phenomenon called depersonalization referred to a feeling of detachment from one's own senses and body) and the surrounding environment (phenomenon known as derealization, a feeling of unreality toward people and objects around) [8]. Moreover, transient episodes of DPD can be found in particular states, such as fatigue and fear [9], as well as in comorbidity with a wide range of psychiatric and neurological conditions [10], such as temporal lobe epilepsy [11], schizophrenia [12], or posttraumatic stress disorder (PTSD) [13]. Physiological and neuroimaging studies have demonstrated neurobiological alterations in the temporal regions in patients with DPD [14], however other studies underlined a fronto-limbic imbalance along with hyperactivity of prefrontal structures and hypoactivation of limbic regions [15]–[17]. A recent line of research suggests that typical symptoms of DPD may be caused by an altered functional connectivity, that may explain the cognitive and emotional disconnection in dissociation [18]. For these reasons, it is possible to hypothesize that brain regions with a key role in sensory integration and sense of self, such as TPJ, may concur to DPD symptomatology. Interestingly, the feeling of disembodiment, as well as the lack of sense of agency or body perceptual distortions and illusions, can be induced experimentally using specific protocol to induce body illusions. For example, the rubber hand illusion paradigm is one of the most well-known procedures to create body distortions and can be used to enhance an illusion of lack embodied self [19]. Amongst others, the most extreme condition in which a depth sense of disembodiment occurs is the Out of Body Experience (OBE), defined as the experience in which a person seems to be awake and to see his body and the world from a location outside his physical body, due to an abnormal sense of spatial unity between the self and the body [20],[21]. Specific experimental methods allow to induce a type of body illusions as a tool to reshapes the boundaries of body and peripersonal representations such as the distinction in which the external between what may or may not be part of the own body (or the distinction between it and external, non-corporeal, objects) [22] and the real perception of one's own body and that of others [23]. One of these experimental methods used to induce body illusion and distortions is the Transcranial Magnetic Stimulation (TMS). TMS is a non-invasive brain stimulation technique allowing the induction of magnetic currents in depth, through the modification of the underlying neural activity [24],[25]. In particular, TMS induces a magnetic field on the scalp, resulting in a depolarization of neuronal tissue and a generation of action potentials [26]. Variables to be considered for a correct use of the TMS are the following: i) stimulation repetition modes: the main stimulation repetition modes include repetitive stimulators (rTMS). rTMS protocols at low frequency (1 Hz) produces a decrement in cortical excitability, while high frequency, usually ranged between 5 and 20 Hz produces its increment [27],[28]. In addition to the conventional rTMS, patterned stimulation protocols have been introduced (i.e., theta burst stimulation). However, the differentiation between decrement and increment of cortical excitability is not well-demarked, since an important degree of inter-individual variability is reported (i.e., inhibitory effect at high frequency rTMS and facilitatory effect at low frequency modulation [29],[30]: for this reason, the concept of functional lesion, the transient and reversible disruption of functionality induced by 1 Hz, has been questioned [31]. It can stimulate up to single pulse in a second [32],[33]; ii) risetime: this term refers to the time taken for a magnetic (or electrical) stimulation pulse to reach its peak amplitude [34]; iii) coil geometry and position: since a focal stimulation could not be delivered, areas that are associated with a specific function are stimulated. The geometry of the coil and its position involve different magnetic fields with specific characteristics [35]; iv) brain depth penetration: it is dependent from coil geometry and size, local anatomy, stimulus strength, and perhaps even gravitational effects on the brain within the skull space [36]; v) safe stimulator design: important the amount of energy involved and the speed with which the energy is delivered [26]. Promising results in adult neurologic and psychiatric disorders are driving active research into transcranial brain stimulation techniques, particularly TMS and transcranial direct current stimulation (tDCS) [37]–[40].

TMS protocols seem to effectively treat a wide range of psychiatric disorders such as mood disorders, schizophrenia, and obsessive-compulsive disorders [41]. In recent times, a few studies have started to investigate the effectiveness of TMS in enhancing an embodied state of consciousness [42],[43].

This systematic review aims to demonstrate the importance of TPJ as “core structure” in functional networks underlying the bodily construction of self, taking into account specific clinical conditions such as DPD and healthy controls subjected to body distortions experimental protocols. Additionally, the present study investigates whether the use of TMS could support the treatment of DPD and other clinical conditions in which depersonalization and derealization are displayed. Finally, we discuss the neural bases underlying the awareness of the body self-awareness.

## 2. Material and methods

This systematic review includes studies of any sham or controlled trial type design which incorporated outcomes concerning the application of TMS on TPJ in functional consciousness disorders such as DPD, but also the experimental induction of illusions (Out-of-Body Experience, Mirror Box Illusion, Rubber end and Body perceiving). Studies were included if the following inclusion criteria were met: (1) participants diagnosed with DPD and healthy participants; (2) stimulation via TMS; (3) TPJ stimulation, (4) human subjects; (5) patients over 18 years old; (6) single or repeated TMS sessions; (7) publications in English language. From the retained selection, we excluded: (1) duplicates; (2) non-human subjects, (3) TMS targeting other areas than TPJ; (4) reviews and (5) irrelevant studies after title/abstract screening. The search strategy was conducted in September 2020 and the publication date was not limited. To identify suitable publications, an online search of the PubMed, Cochrane Library, Web of science and Scopus, databases was performed using specific search terms “TMS” AND “temporoparietal junction” AND “self”, “TMS” AND “temporoparietal junction” AND “awareness”, “TMS temporoparietal junction” AND “depersonalization”, “TMS” AND “temporoparietal junction” AND “derealisation”, “TMS temporoparietal junction” AND “body”. Three independent reviewers (GO, VC, DB) performed the screening of the articles based on title and abstract. Duplicates were checked and removed using Mendeley desktop reference manager (http://www.mendeley.com). A visual independent check of duplicates was performed to ensure the complete removal of duplicates. Additionally, review articles and the references section of each article included in the study was checked to find additional hits satisfying the inclusion criteria according to the purpose of this review.

## 3. Results

Our search identified a total of 108 records through multiple databases searching and 1 additional record identified through other sources (Table 1).

**Figure 1.**
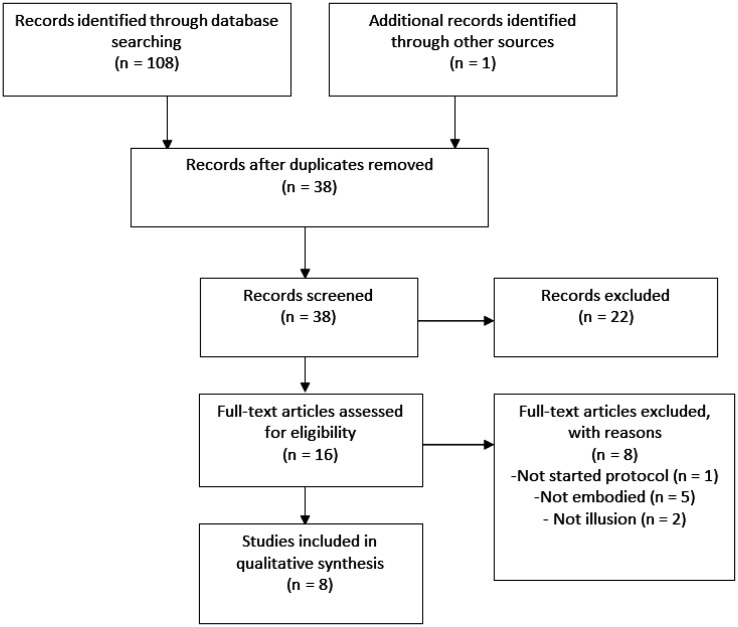
Prisma flow chart of study selection.

After duplicates removal, 38 hits were screened (Figure 1). After title and abstract reading, we retained a total of 16 records for the assessment of eligibility. According to our inclusion criteria, we retained 8 studies for a qualitative synthesis.

**Table 1.** Search strategy, databases, date of access and MESH terms.

Database	Date of Search	MeSH Terms					Results
		TMS	TMS	TMS	TMS	TMS	
		temporoparietal junction self	temporoparietal junction and awareness	temporoparietal junction and depersonalization	temporoparietal junction and derealization	temporoparietal junction body	
Pubmed	16/09/2020	14	5	3	2	7	31
Cochrane Library	16/09/2020	11	4	3	1	0	19
Web of Science	16/09/2020	16	4	0	1	12	33
Scopus	16/09/2020	11	4	3	0	7	25
						Total Results	108

The selected articles were classified into two sub-categories: the first containing the application of TMS on TPJ for reducing the feeling of disembodiment for therapeutic purposes in DPD patients (4 studies); the second concerning the application of TMS on TPJ for inducing the feeling of disembodiment to neurophysiological and theoretical purposes (illusion) (4 studies).

### 3.1. TMS in DPD

We investigated the effects of TMS targeting TPJ in subjects with DPD or subjects with numbness, feeling of unreality, feeling of detachment from oneself/surrounding (Table 2). Specifically, all the studies used repetitive transcranial magnetic stimulation (rTMS).

**Table 2.** Experimental Illusion for disembodiment induction.

Author	S Sample (N)	Stimulation	Coil	Frequency	Pulses	Session	Measures	Procedure	Coil Position/Method	Outcome
Mantovani et al., 2011 [43]	N = 12	rTMS (Magstim Super-rapid stimulator)	Fo8 (70 mm)	1 Hz	800 per session	3 weeks plus 3 weeks (30–42 sessions; not stated)	CADSS, HDRS, HARS, CGI-S, CDS, DES, BDI–II, Zung-SAS, PGI	Baseline and after each week	rTPJ or lTPJ, between T4/P4 and T3/P3 respectively, according to 10–20 EEG System	After 3 weeks, 6 out of 12 patients responded. Five responders have received 3 more weeks of rTPJ-rTMS showing 68% DPD symptoms improvement
Christopeit et al., 2014 [44]	N = 12	rTMS (Magstim Super-rapid stimulator)	Fo8 (70 mm)	1 Hz	800 per session	3 weeks plus 3 weeks (30–42 sessions; not stated)	CADSS, HDRS, HARS, CGI-S, CDS, DES, BDI–II, Zung-SAS, PGI	Baseline and after each week	rTPJ or lTPJ, between T4/P4 and T3/P3 respectively, according to 10–20 EEG System	Reduction in anomalous body experiences, alienation from surroundings, anomalous subjective recall and emotional numbing
Jay et al., 2014 [45]	N = 37	rTMS (Magstim Rapid2)	NA	1 Hz	900	1	CDS, BDI, BAI, DES, skin conduction, subjective arousal	Pre/post rTMS, phone interview 24 h post rTMS	rVLPFC, neuronavigated	Increase of electrodermal capacity. DD patients showed increased SFs of skin conductance post rTMS. Both rVLPFC and rTPJ-rTMS showed a reduction in DDS
Wulf et al., 2019 [46]	N = 4	rTMS	NA	Cond.1 = 1 Hz Cond.2 = 1 Hz	Cond.1 = 1800 pulses Cond.2 = 900 pulses	15 rTMS stimulation over three weeks	NA	Baseline and after 6-week	Cond.1 = temporoparietal junction (TPJ) Cond.2= right ventrolateral prefrontal cortex (rVLPFC)	Reduction of symptoms of rTMS over TPJ Reduction of symptoms in 6-week-FU rating compared to baseline (TMS over rVLPFC)

Notes: BAI: Beck Anxiety Inventory; BDI-II: Beck Depression Inventory; BS: between subject; CADSS: Clinician-Administered Dissociative States Scale; CBT: cognitive- behavioral-therapy; CDS: Cambridge Depersonalization Scale; CGI-S: Clinical Global Impression-Severity; Cond: Condition; DDS: Depersonalization-derealization-syndrome; DES: Dissociative Experiences Scale; DPD: depersonalization disorder; EEG: Electroencephalogram; FU: follow-up; HARS: Hamilton Anxiety Rating Scale; HDRS: Hamilton Depression Rating Scale; lTPJ: left temporoparietal junction; NA: not available; PGI: Patient Global Impression; rIPS: right intraparietal sulcus; rTMS: transcranial magnetic stimulation; rTPJ: right temporoparietal junction; rVLPFC: right ventrolateral prefrontal cortex; SFs: spontaneous fluctuations; TPJ: temporoparietal junction; Zung-SAS: Zung- Self Administered Scale.

**Table 3.** Experimental Illusion for disembodiment induction.

Author	S Sample (N)	Stimulation	Coil	Frequency	Pulses	Session	Measures	Procedure	Coil Position/Method	Outcome
Blanke, 2005 [42]	N = 11	rTMS (Magstim Rapid stimulator)	Fo8 (70 mm)	1 Hz	900	4 (2 sessions per day for 2 days)	Average RTs of correct responses	Pre and Post rTMS	rEBA and TPJ in different sessions	Selective activation of the TPJ (at 330–400 ms after stimulus onset) during the imagery task that resemble OBE
Tsakiris et al., 2008 [22]	N = 10	Single pulse erTMS (2T Magstim 200)	Fo8 (70 mm)	ER	160. One per trial (350 ms after stimuli)	1	Proprioceptive drift (cm)	Each trial, post stimulus and TMS (after 3350 ms)	rTPJ (Mean MNI = 63.4, −50, 22.7). TPJ defined as intersection of SMG, AG and STG	TMS applied over rTPJ influenced proprioceptive drifts
Papeo et al., 2010 [47]	N = 14	Single pulse erTMS (Magstim 200)	Fo8 (70 mm)	ER	One per trial (350 ms post stimuli)	1	Accuracy and RT on congruent and incongruent trials	Within 3 s of each stimulus application	rTPJ (Mean MNI: 63.4, −50.0, 22.7; intersection of SMG, AG and STG), neuronavigated based on prior MRI data	Poor accuracy for digit 4 compared to digit 3, particularly when incongruent. Improved accuracy for digit 4 incongruent trials Reduced conflicting visual information after TMS
Cazzato et al., 2014 [23]	N = 66	rTMS (Magstim Rapid)	Fo8 (70 mm)	1 Hz	900	3 (TPJ, EBA, no-TMS)	Body distortion score; object distortion score	Pre- and post-rTMS	rEBA, rTPJ (Talairach = 63, −50, 23)	The outcomes detected showed that the stimulation of rTPJ leads to an overestimation bias when the subjects had to make judgments about other people' body. This effect is more consistent in right EBA than in left EBA, greater in women than in men

Notes: AG: angular gyrus; EBA: extrastriate body area; no-TMS: not TMS; MNI: Montreal Neurological Institute coordinate system; OBE: out of body experience; rEBA: right extrastriate body area; rTMS: transcranial magnetic stimulation; rTPJ: right temporoparietal junction; RTs: reaction times; SMG: right supramarginal gyrus; STG: superior temporal gyrus; TPJ: temporoparietal junction.

Mantovani et al. [43], investigated the effect of inhibitory low frequency rTMS over the right TPJ (rTPJ) for three weeks, the right TPJ of 12 outpatients was targeted. During the following three weeks, the six outpatients who responded positively were stimulated on the same targeted area. In this regard, an improvement of 68% (F = 8.81; df = 6; p = 0.000) in DPD symptoms were shown. Moreover, partial/non-responders were stimulated on the left TPJ (lTPJ), but no significant improvement of DPD symptoms was shown. Based on the results obtained by Mantovani et al. [43], Christopeith and colleagues [44] conducted a retrospective analysis on data excerpts from the study. They have divided the scores derived by the Cambridge Depersonalization Scale (CDS) on the following symptoms clusters: reductions in anomalous body experiences, alienation from surroundings, emotional numbing, and anomalous subjective recall. After three weeks stimulation of rTPJ, significant reductions of anomalous body experiences (50% improvement; F = 4.7; df = 3; p = 0.008) and anomalous subjective recall (22% improvement; F = 3.1; df = 3; p = 0.041) were detected. Whereas after six weeks of rTMS, an additional significant reduction in anomalous body experiences (76% improvement; F = 5.1; df = 6; p = 0.002) and alienation from the surrounding environment (54% improvement; F = 3.1; df = 6; p = 0.023) were found. Moreover, a clinical, but non-significant improvement in emotional numbing (52% reduction; F = 2.4; df = 6; p = 0.057) and anomalous subjective recall were found (57% improvement; F = 1.9; df = 6; p = 0.115).

Jay and colleagues [45] tested the effect of 1 Hz rTMS targeting ventrolateral prefrontal cortex (VLPFC) or TPJ on patients affected by medication resistant DPD. To this purpose, measures maximum skin conductance capacity, as well as the CDS for the assessment of symptomatology were employed. Moreover, a secondary outcome included spontaneous fluctuations (SFs) and event-related skin conductance responses. Results shown a reduction of symptoms after both conditions (t = −2.2; df = 19; p = 0.04), but only after rTMS targeting VLPFC, an increase of electrodermal capacity, namely maximum skin conductance deflections, was reported (t = −2.2; df = 7; p < 0.05). No change in event-related electrodermal activity was detected after rTMS protocol.

Recently, Wulf et al. [46] adopted the protocol used by Mantovani et al. [44] for the stimulation of right VLPFC, whereas for the stimulation of right TPJ the authors used the protocol employed by Jay et al. [45]. At the same time, all the patients received Cognitive Behavioral Therapy (CBT) comprising interventions such as mindfulness, confrontation, muscle relaxation and sports. After six weeks, a follow up evaluation highlighted the ameliorating effects of combined CBT-TMS treatment on DPD.

### 3.2. TMS protocol for inducing disembodiment

The use of TMS on TPJ in healthy volunteers, can enhance the feeling of disembodiment, altered the boundaries of body and the capacity for proprioception and lead to alterations in the evaluation of multisensory conflicts and in the evaluation of one's own or others' body (Table 3).

Blanke et al. [42], investigated the role of TMS over TPJ to induce OBE in a group of 11 healthy volunteers. Using evoked potential mapping, authors showed the selective activation of the TPJ at 330–400 ms after stimulus onset when healthy volunteers imagined themselves in the position and visual perspective that generally are reported by people experiencing spontaneous OBEs. In this study, it has been found that TMS over TPJ impaired mental transformation of one's own body in healthy volunteers (F = 24.4; p = 0.003) relative to TMS delivered over a control site located on the intraparietal sulcus (IPS) (F = 2.16; p = 0.19).

In order to investigate the role of the rTPJ in preserving a coherent body ownership, Tsakiris and colleagues [22] tested the effect of proprioceptive judgment (drift) after a four-block trial in which rTPJ or the vertex (as control site) were stimulated 350 ms after visuo-tactile synchronous stimulation, while the subjects were viewing either a rubber hand or neural object. Additionally, participants performed two further blocks viewed either the rubber hand or neutral object while receiving asynchronous stroking, without TMS stimulation. The results of the study showed that TMS over the rTPJ decreases the incorporation of the rubber hand into the mental representation of one's own body (t = 4.67; p < 0.05; two-tailed). On the contrary, authors found an increment incorporation of a neutral object (t = 2.55, p < 0.05, two-tailed).

To investigate the role of rTPJ in intersensory conflicts, Papeo et al. [47] used a single pulse TMS delivered 350 ms after visuotactile stimulation in the mirror-box illusion. Results showed a reduced accuracy in localizing the left touch for the ring finger rather than the middle finger in the incongruent trials (F = 8.250; p < 0.02). After TMS, an improvement of accuracy indexes for ring finger in incongruent trials was found, suggesting the role of rTPJ in detecting (p = 0.01), rather than resolving, multisensory conflicts.

The role of TPJ and extrastriate body area (EBA; located at the posterior inferior temporal sulcus/middle temporal gyrus) in perceiving one's own body and others body has been investigated by Cazzato and colleagues [23] in which a body distortion technique combined with rTMS stimulation was used. Participants had to judge the size readjustments on images of their own or other bodies, both from a subjective or intersubjective perspective. The outcomes detected showed that the stimulation of rTPJ leads to an overestimation bias when the subjects had to make judgments about other people' body (F = 4.009; p = 0.025). This effect was more consistent in right EBA than in left EBA, greater in women than in men. A strong lateralization of the right hemisphere of the active role of the EBA in aesthetic judgments is therefore observed in women, but less in men.

## 4. Discussion

This review analysed the efficacy of TMS targeting TPJ in patients with DPD and the illusion of disembodiment induced in healthy subjects. TPJ is hypothesized to be a core hub for an embodied concept of consciousness. TMS stimulation on TPJ in patients with symptoms of depersonalization and derealization leads to an improvement in the severity of the symptoms. On the other hand, the application of the TMS on TPJ in healthy patients can induce phenomena of illusion perception of self. The studies included in this review showed a significant reduction of symptoms in DPD patients treated with inhibitory low frequency rTMS over rTPJ for three weeks [43],[45], as well as a notable reduction of anomalous body experiences [44]. A six-week CBT-TMS combination treatment in DPD patients resulted in a significant improvement in symptoms such as depersonalization and derealization [46]. Likewise, the application of TMS on TPJ in healthy volunteers, through specific experimental procedures, may induce body illusions. For instance, it was demonstrated that the application of TMS on TPJ impaired mental transformation of one's own body [42], proprioceptive abilities [22] and the resolution of intersensory conflicts [47]. Furthermore, Cazzato et al. [23] observed difference between man and woman in the hemispheric asymmetry of EBA region, in the aesthetic processing of human bodies. Women have more dominance of the right hemisphere than men.

Taken collectively, the results of the considered studies showed evidence of the positive effects of TMS on TPJ or between temporal and parietal regions (in particular, T4/T3 and T3/P3) in patients with a diagnosis of DPD. The results need to be implemented with further scientific evidence. Some studies pointed out a large-scale neural integration as the basis of a correct perception of the body self [18]. In fact, neuroimaging research has shown a fronto-limbic imbalance in DPD subjects, with prefrontal hyperactivation and amygdaloidal hypoactivation [48],[16], and a lower functional activity in temporal regions [49]–[51]. A recent work by Sierk et al. [51] observed significantly less fractional anisotropy between the right middle temporal gyrus (MTG) and the right supramarginal gyrus (SMG) in subjects with DPD, as found in other studies [52],[53]. A lower structural connectivity was also observed between the left temporal pole and the left superior temporal gyrus. In addition, the severity of dissociative symptoms is negatively correlated with the fractional anisotropy values of this connection.

SMG is a fundamental region for the integration of sensory information, due to its involvement in receiving afferents from visual, auditory, somatosensory and limbic structures, and has been associated with cross-modal spatial attention [54]. MTG appears to be important for conceptual processing [55],[56] and transmodal integration [57],[58]. Furthermore, the left temporal pole plays an important role in creating associations between different functions [59] and the left superior temporal gyrus is involved in auditory processing [60]. Partial and temporal regions are partially included in TPJ, so it is possible to hypothesize their functional involvement in the integration of information for a correct perception of the body self. As evidence of what has been discussed, we have shown above that TMS stimulation of these regions leads to an improvement in the symptoms of DPD [43]–[46]. Similarly, a stimulation of TPJ in healthy subjects induces bodily illusion. To date, it remains unclear how the fronto-limbic imbalance, that is a core feature in patients with DPD, is associated with dysfunction of the TPJ.

In the context of TMS stimulation protocol targeting medial prefrontal cortex (mPFC), Gruberger and colleagues [61] also reported dissociative and detachment phenomena after the induction of the functional lesions: indeed, the use of large H coil used by the authors could have produced an alteration of the ongoing sense of self by indirectly involving the TPJ. It is worth nothing that both TPJ and mPFC are functionally connected within Default Mode Network, that play a pivotal role in self-introspective and self-mentalization activity [62].

It is important to underline that depersonalization and derealization symptoms are not exclusively present only in the DPD, but also in multiple psychopathological disorders such as anxiety disorders, mood disorders, post-traumatic stress disorder and epilepsy. Moreover, transient episodes of depersonalization and derealization are often experienced by non-clinical populations. For these reasons, the use of methods such as TMS on TPJ can prove to be a valid treatment tool for the improvement of many clinical disorders.

## 5. Conclusion

In conclusion, there are several limitations to be addressed regarding the correct assessment of the role of the TPJ related to self-perception. TPJ is a network with a wide range of afferences; therefore, its position, structure and parcellation lead to a difficult comprehension of its functions. Other challenges encountered were the following: low number of studies using TMS on TPJ in patients with DPD; small sample size; moderate heterogeneity of paradigms between studies; limited data reported and limited statistical significance.

Furthermore, we were not able to define the number of TMS sessions sufficient to induce effects on symptoms. Future studies, should use protocols that include control groups and follow-up sessions, more reliable measures and both qualitative and quantitative assessments.

To our knowledge, this review is the first focusing on the use of TMS on TPJ in DPD. This review shows the relationship between DPD with TPJ and the efficacy of TMS in the treatment of these disorders. Furthermore, this review is the first focusing on use of TMS on TPJ for inducing disembodiment. These data are a first step towards a greater understanding of possible treatments to be used in embodiment disorders. It is important to highlight that despite the promising approaches investigated so far, due to the challenges encountered, conclusions on the effectiveness of the reported methods are premature.
